# The association between energy-adjusted dietary inflammatory index and metabolic syndrome and its mediatory role for cardiometabolic diseases: a prospective cohort study

**DOI:** 10.3389/fnut.2024.1429883

**Published:** 2024-08-05

**Authors:** Hossein Pourmontaseri, Matin Sepehrinia, Mohammad Shafi Kuchay, Mojtaba Farjam, Farhad Vahid, Azizallah Dehghan, Reza Homayounfar, Mohammad Mehdi Naghizadeh, James R. Hebert

**Affiliations:** ^1^Student Research Committee, Fasa University of Medical Sciences, Fasa, Iran; ^2^Medanta The Medicity, Gurugram, Haryana, India; ^3^Noncommunicable Diseases Research Center, Fasa University of Medical Sciences, Fasa, Iran; ^4^Nutrition and Health Research Group, Department of Precision Health, Luxembourg Institute of Health, Strassen, Luxembourg; ^5^National Nutrition and Food Technology Research Institute (WHO Collaborating Center), Faculty of Nutrition Sciences and Food Technology, Shahid Beheshti University of Medical Sciences, Tehran, Iran; ^6^Department of Epidemiology and Biostatistics, Arnold School of Public Health, University of South Carolina, Columbia, SC, United States; ^7^South Carolina Statewide Cancer Prevention and Control Program, University of South Carolina, Columbia, SC, United States

**Keywords:** metabolic syndrome, inflammation, dietary inflammatory index, diabetes, myocardial infarction, stroke

## Abstract

**Background:**

Metabolic syndrome (MetS) is a collection of medical conditions that elevate the chance of cardiovascular disease. An unhealthy diet is a major risk factors for MetS through different mechanisms, especially systemic chronic inflammation.

**Objective:**

This study aimed to investigate the effect of dietary inflammatory potential on MetS incidence and the role of MetS in the association between Energy-adjusted dietary inflammatory index (E-DII) and cardiometabolic diseases.

**Methods:**

In this prospective cohort study, 10,138 participants were recruited. All participants were divided into MetS or non-MetS groups based on the Adult Treatment Panel III criteria. The E-DII was used to assess the inflammatory potential of diet. After excluding the participants with MetS at baseline, 2252 individuals were followed for 5 years (longitudinal phase), and the effect of E-DII on MetS incidence was investigated using logistic regression models (*p*-value <0.05).

**Results:**

The cohort’s mean age (45.1% men) was 48.6 ± 10.0 years. E-DII ranged from −6.5 to 5.6 (mean: −0.278 ± 2.07). Higher E-DII score had a 29% (95%CI: 1.22–1.36) increased risk for incidence of MetS and its components during five-year follow-up. Also, E-DII was significantly associated with the prevalence of MetS (OR = 1.55, 95%CI: 1.51–1.59). Among MetS components, E-DII had the strongest association with waist circumference in the cross-sectional study (OR = 2.17, 95%CI: 2.08–2.25) and triglyceride in the longitudinal study (OR = 1.19, 95%CI: 1.13–1.25). The association between E-DII and MetS was consistent in both obese (OR = 1.13, 95%CI:1.05–1.21) and non-obese (OR = 1.42, 95%CI: 1.27–1.60) individuals and stronger among non-obese participants. Additionally, MetS mediated the association between E-DII and hypertension, diabetes, and myocardial infarction.

**Conclusion:**

In conclusion, a pro-inflammatory diet consumption is associated with a higher risk of MetS and its components. Furthermore, a pro-inflammatory diet increases the risk of cardiometabolic diseases. The higher E-DII had a stronger association with MetS, even among normal-weight individuals.

## 1 Introduction

Metabolic Syndrome (MetS) is a collection of factors that contribute to a higher likelihood of developing cardiovascular disease (CVD). These conditions are represented by abdominal obesity (assessed by measurement of waist circumference, WC), impaired glucose, high blood pressure, and dyslipidemia, significantly increased levels of triglycerides (TG), and low high-density lipoprotein cholesterol (HDL-C) levels (1). Each of the five MetS components has an independent association with the progression of non-alcoholic fatty liver disease, coronary artery diseases, and stroke, but combining them together in MetS increased their prognostic properties (2). Since all the components of MetS respond to lifestyle changes, it remains the safe and cost-effective treatment for the prevention and treatment of MetS and its components (3, 4).

To date, several successful dietary interventions have been developed to control MetS and prevent its related complications, such as nonalcoholic fatty liver disease, cardiovascular diseases, and diabetes (5). Most of the present dietary recommendations focus on lowering energy intake (6). Although previous dietary recommendations achieved some success in controlling MetS, recent studies showed that diets with anti-inflammatory properties, such as the Mediterranean diet, had more efficiency in the management of MetS and further complications (7).

Nutrition influences the progression of MetS by different mechanisms. Chronic inflammation and prolonged increase of plasma pro-inflammatory factors are essential conditions that provoke, accelerate, and complicate MetS-related disorders such as CVD (8). The Dietary Inflammatory Index (DII) is one of the best tools for assessing the diet’s inflammatory potential, revealing the influence of daily nutrition on plasma inflammatory biomarkers (9). Till now, DII has been updated several times. In 2019, Energy-adjusted DII (E-DII) was introduced to achieve an accurate calculation by adjusting the energy intake and providing a more generalizable index (10). Recent studies showed that higher DII was associated with risk for CVD, MetS, and mortality (11, 12), while others indicated that this association was insignificant (13, 14).

Previous studies have evaluated the association between the diet’s inflammatory potential and MetS and its components. Still, there are controversies in the case of the association between DII and MetS as some studies found a significant positive association, but some studies failed to find a significant association. Also, prospective studies about the association between E-DII and MetS are scarce (15). MetS is a strong predictor of cardiometabolic diseases. Previous studies showed that the pro-inflammatory diet was associated with a greater chance of cardiometabolic diseases such as diabetes and CVD (16). Recent research revealed that more than 30% of Iranian adults had MetS, and cardiovascular disease is the most common cause of mortality in Iran (17). Therefore, the present study aimed to investigate the effect of E-DII on MetS and its related diseases in an Iranian population. Furthermore, this study aimed to evaluate the role of MetS in the association between E-DII and cardiometabolic diseases.

## 2 Materials and methods

### 2.1 Study design and population

**Figure 1:**
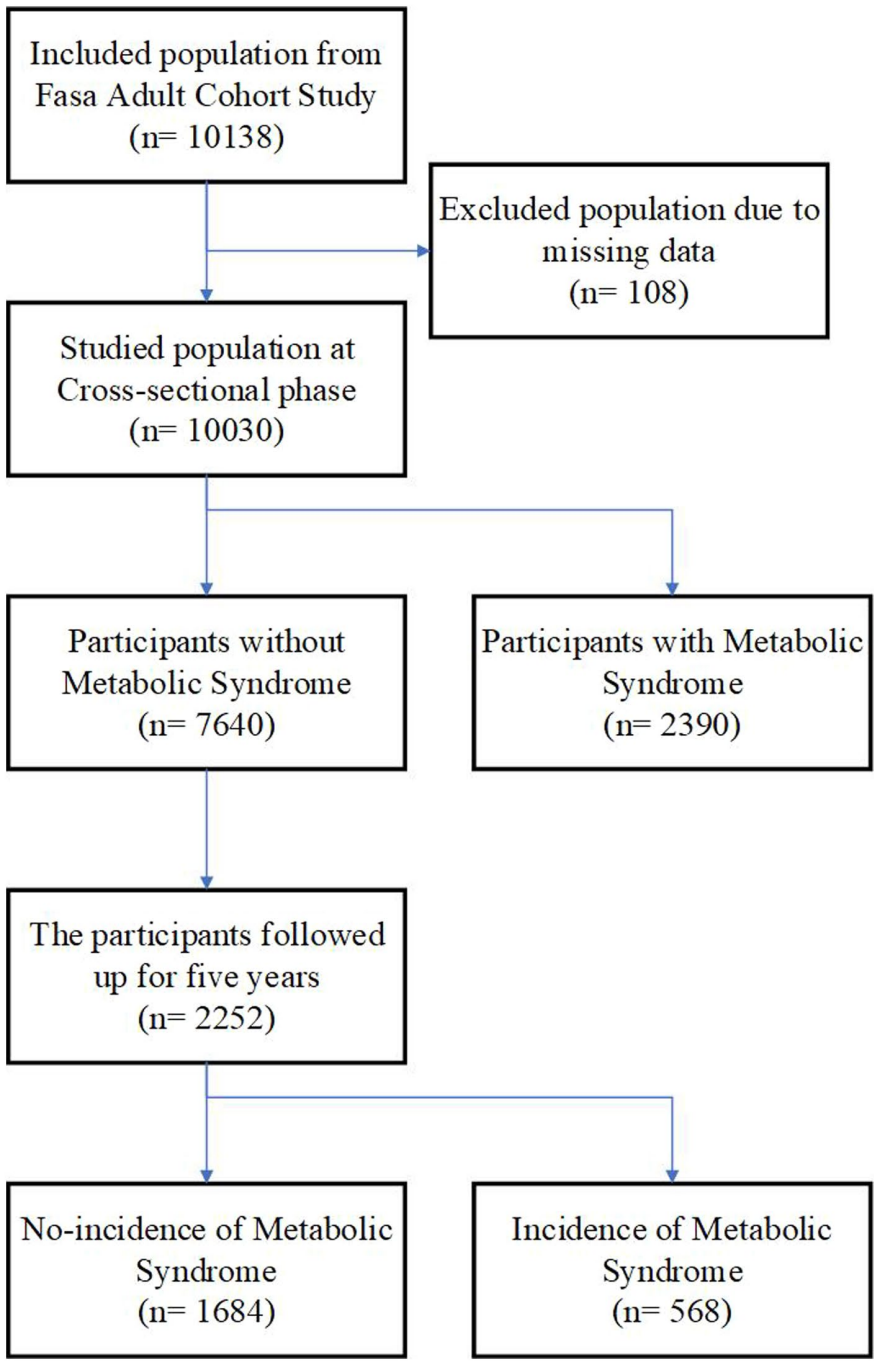
The flow chart of study.

This prospective study was conducted on the Fasa Adult Cohort Study (FACS) with 10,138 adult participants aged 35 to 70 years old from Sheshdeh, Fasa, Iran. FACS is one of the population-based cohorts of Prospective Epidemiological Research Studies in Iran (PERSIAN), national-wide studies investigating the prevalent noncommunicable diseases, such as CVD, diabetes, MetS, and non-alcoholic fatty liver diseases in Iran. The participants fulfilled written consent before being included in FACS, and comprehensive surveys including sociodemographic, health status, anthropometrics, and food frequency questionnaire (FFQ) were collected from them. The first data collection phase was completed in 2016, and the plasma, urine, hair, and nail samples were stored in the data bank of FACS (18–20). After excluding the participants with missing data, the remaining participants were categorized as MetS and non-MetS, and the association between the E-DII and MetS and further MetS-associated diseases was investigated (Figure 1). Also, the participants were separated into low (<25 kg/m2, *n* = 1,179) and high (>5 kg/m2, *n* = 1,037) body mass index to investigate the association of E-DII and MetS among normal and obese patients.

### 2.2 Measurements

The complete procedure of data collection was explained elsewhere (18). The sociodemographic characteristics, including age (year), gender (men, women), marital status (single, married, widow, and divorced), occupation (having a job or not), education (no education, primary school, secondary school, university), and physical activity (metabolic equivalent of tasks) activity was included from data bank of FACS. Abusing cigarettes (active smokers or not), alcohol (regular consumption or not), and opium (regular consumption or not). Also, the health status of participants, including having a myocardial infarction (MI), diabetes, hypertension, and stroke, was detected based on self-report, specialist diagnosis, consuming related medication, or recorded medical documents of participants. The anthropometric characteristics of each participant, including body mass index (kg/m^2^), hip circumference (cm), waist circumferences (cm), wrist circumference (cm), and systolic and diastolic blood pressure (mmHg) were measured and reported based on International Units. TG (mg/dL), fasting blood glucose (mg/dL), and HDL-C (mg/dL) were assessed using the plasma sample gathered at phase one and stored in the data bank of FACS (21).

### 2.3 Assessment of energy-adjusted dietary inflammatory index

A semi-quantitative 125-item food frequency questionnaire (FFQ) was applied to assess the quality and quantity of food consumed by the participants of FACS. The daily, weekly, monthly, and annual intake of Iranian food over the past year was asked participants to achieve a modified assessment of nutrient consumption appropriate for the Iranian population (22). The FFQ was based on the Willet format questionnaire and altered to fit Iranian dietary habits (23) The United States Department of Agriculture (USDA) determined the portion sizes for each food item (21). Further information regarding the validity and specifics of the FFQ is discussed elsewhere (24).

In this study, EDII was calculated based on FFQ data. The EDII is a quantitative measurement of the inflammatory potential of diet, which assesses the consumption of 45 dietary parameters. These parameters were divided into three groups: anti-inflammatories (scored −1), pro-inflammatory (scored +1), and no inflammatory effect (scored 0). Complete detailed coefficients and formulation of EDII were explained elsewhere (25). From 45 dietary parameters of DII, 31 parameters were included (Vitamin B12, Vitamin B6, Beta Carotene, Caffeine, Carbohydrate, Cholesterol, Energy, Total fat, Fiber, Folic acid, Garlic, Fe, Mg, MUFA, Niacin, n-3 Fatty acids, n-6 Fatty acids, Onions, Proteins, PUFA, Riboflavin, Saturated fat, Se, Thiamin, Trans fat, Vitamin A, Vitamin C, Vitamin D, Vitamin E, Zn, and peppers). The dietary intake was calculated for each participant. To calculate the z-score for each individual, the standard global mean was subtracted the standard global mean from the dietary intake of each parameter. Then, it is divided by the international standard deviation. Then, obtained values were converted to centered percentile scores to minimize the effect of right skewing- the proportion centered by doubling and subtracting 1. Then, we multiplied the centered percentile value by the inflammatory effect score of each food parameter based on Shivappa et al.’s study. All scores were summed to obtain the overall DII score for each participant (25). To adjust DII for energy, all dietary parameters were converted to the food intake per 1,000 calories (26).

### 2.4 Assessment of MetS and cardiometabolic diseases

The MetS was assessed based on Adult Treatment Panel III, which is appropriate for screening MetS with a high sensitivity in large populations (27). In this definition, the individuals who pass three or more inclusion criteria are called MetS. The five include: 1. High waist circumference (men: >102 cm, women: > 88 cm); 2. High blood pressure (systolic >130 mmHg or diastolic >85 mmHg); 3. low HDL-C (<40 mg/dL for men and < 50 mg/dL for women); 4. High TG (> 150 mg/dL); 5. High fasting blood sugar (>100 mg/dL). Hypertension (HTN), stroke, MI, and diabetes were based on the participants’ medical records of electronic documents in FACS (21).

### 2.5 Statistical analysis

The data from the present study was recorded and analyzed in IBM SPSS Version 23 (Armonk, NY: IBM Corp.). The qualitative and quantitative variables were reported as frequency (percent) and mean (standard deviation). The independent t-test and chi-square were used to compare the mean of quantitative and frequency of qualitative variables among the MetS and the non-MetS groups. The factors with considerable differences (*p*-value <0.20) were included as potential covariates (age, gender, education, occupational status, physical activity, socioeconomic status, smoking, opium, and alcohol) for multivariate analysis. Then, Wald’s logistic regression model was applied to investigate the final significant covariates (age, gender, education, physical activity, socioeconomic status, and smoking). The crude and adjusted association of E-DII with MetS and related diseases was analyzed using logistic regression. The significant level was considered as *p*-value <0.05.

#### Ethics

All steps of this prospective study followed the Helsinki Declaration and were confirmed by the Ethics Committee of Fasa University of Medical Sciences (IR.FUMS.REC.1401.162). Each participant of the FACS fulfilled a written informed consent and was aware of the aim of FACS (14). The data of the present study were extracted from the FACS database.

## 3 Results

In the first cross-sectional phase, 10,030 individuals (mean age of 48.6 ± 10.0 years), including 4,523 (45.1%) men. The mean of E-DII was −0.278 ± 2.07, ranging from −6.5 to 5.6. Approximately 23.8% (*n* = 2,390) of participants had MetS in phase one. Table 1 shows the baseline characteristics and compares them between participants with and without MetS. The female participants significantly had a higher rate of MetS. The prevalence of hypertension, MI, diabetes, and stroke was significantly higher in the MetS group. Also, the participants with MetS had lower education, physical activity, and socioeconomic status. Interestingly, smoking, opium, and alcohol consumption were significantly lower among individuals with MetS (*p*-value was <0.001 for all). Furthermore, participants with MetS consumed lower amounts of anti-inflammatory nutrients such as vitamins, magnesium, selenium, fiber, and caffeine. However, there was no significant difference in consumption of zinc and n6-fatty acids between participants with and without MetS (Supplementary Table S1).

**Table 1:** Comparing the baseline characteristics of the studied population among non-MetS and MetS groups (*n* = 10,030).

Variable	Subgroup	Total	Non-MetS (*n* = 7,640)	MetS (*n* = 2,390)	*P* value
Age (years)		48.6 (10.0)	47.7 (9.4)	51.6 (9.3)	<0.001
Gender, Male		4,523 (45.1)	3,998 (52.3)	525 (22.0)	<0.001
Diabetes		1,232 (12.3)	535 (7.0)	697 (29.2)	<0.001
Hypertension		2010 (20.0)	863 (11.3)	1,147 (48.0)	<0.001
Myocardial infarction		169 (1.7)	103 (1.3)	66 (2.8)	<0.001
Stroke		122 (1.2)	66 (0.9)	56 (2.3)	<0.001
Education	No education	4,595 (45.8)	3,178 (41.6)	1,417 (59.3)	<0.001
	Primary school	3,270 (32.6)	2,576 (33.7)	694 (29.0)	
	Secondary school	1935 (19.3)	1,683 (22.0)	252 (10.5)	
	University	230 (2.3)	203 (2.7)	27 (1.1)	
Occupied		4,977 (49.6)	3,305 (43.3)	1,672 (70.0)	<0.001
Physical activity (METs)		41.5 (11.3)	42.4 (12.0)	38.6 (8.3)	<0.001
Socioeconomic status (Assert index)		−0.001 (2.118)	0.036 (2.16)	−0.121 (1.98)	<0.001
Smoking		1926 (19.2)	1746 (22.9)	180 (7.5)	<0.001
Opium		2094 (20.9)	1888 (24.7)	206 (8.6)	<0.001
Alcohol		204 (2.0)	184 (2.4)	20 (0.8)	<0.001
Body mass index (Kg/m^2^)		25.7 (4.8)	24.7 (4.5)	28.9 (4.4)	<0.001
Waist circumference (cm)		93.1 (11.8)	90.4 (11.0)	102 (9.8)	<0.001
Wrist circumference (cm)		16.7 (1.3)	16.6 (1.3)	17.1 (1.4)	<0.001
Hip circumference (cm)		99.6 (8.8)	98.2 (8.4)	104 (8.6)	<0.001
TG (mg/dl)		132.0 (82.6)	114 (62.0)	190 (109)	<0.001
HDL-C (mg/dl)		51.0 (15.9)	52.7 (16.3)	45.5 (13.1)	<0.001
Fasting blood sugar (mg/dl)		92.6 (29.5)	87.3 (18.5)	110 (45.6)	<0.001
Diastolic blood pressure (mmHg)		74.7 (12.0)	72.9 (11.2)	80.3 (12.3)	<0.001
Systolic blood pressure (mmHg)		111 (18.4)	108 (16.9)	121 (19)	<0.001
E-DII		−0.28 (2.07)	−0.68 (1.97)	1.01 (1.86)	<0.001

The quantitative and qualitative variables were compared using the independent T-test and the chi-square tests. MetS, Metabolic syndrome; MET, Metaoblic Equivalent of Task; TG, Trigeliceride; HDL-C, High-Density Lipoprotein cholesterol; E-DII, Energy-adjusted dietary inflammatory index. (*N*, % or Mean, Standard Deviation).

As presented in Table 2, the E-DII was significantly associated with MetS and its components. These results were consistent after adjusting the model for confounding factors. The highest association was observed between E-DII and high WC as an indicator of obesity in MetS.

**Table 2:** Unadjusted and adjusted association of energy-adjusted dietary inflammatory Index (continuous) as a predictor of the prevalence of metabolic syndrome (*n* = 2,390) and its five components separately as outcome variables in the logistic regression model (*n* = 10,030).

Variable	Unadjusted		Adjusted*	
	OR (95%CI)	*P* value	OR (95%CI)	*P* value
MetS	1.55 (1.51, 1.59)	<0.001	1.55 (1.51, 1.59)	<0.001
Low HDL-C	1.14 (1.12, 1.16)	<0.001	1.15 (1.12, 1.17)	<0.001
High BP	1.21 (1.19, 1.24)	<0.001	120 (1.18, 1.23)	<0.001
High TG	1.51 (1.47, 1.54)	<0.001	1.51 (1.47, 1.54)	<0.001
High FBS	1.23 (1.20, 0.126)	<0.001	1.21 (1.17, 1.24)	<0.001
High WC	1.51 (1.47, 1.54)	<0.001	2.17 (2.08, 2.25)	<0.001

*Logistic regression was used to investigate the adjusted and unadjusted association of the Energy-adjusted Dietary Inflammatory Index with the prevalence of Metabolic Syndrome. Adjusted for age, gender, education, physical activity, socioeconomic status, smoking. MetS, metabolic syndrome; HDL-C, high-density lipoprotein cholesterol; BP, blood pressure; TG, triglyceride; FBS, fasting blood sugar; WC, waist circumference.

After excluding the participants with MetS at baseline, 568 out of 2,252 participants were diagnosed with metabolic syndrome during 5 years of follow-up. Table 3 shows the association of E-DII with the five-year incidence of MetS, OR 1.55 and 95%CI: (1.51, 1.59), and its components after excluding the participants who had MetS in the baseline. The participants with higher E-DII had a significantly higher chance of incidence of MetS and its components. Also, these associations remain significant after adjusting for age, gender, physical activity, and opium. The association of E-DII with MetS and its component was more significant among the participants with normal body mass index compared with obese participants (Table 4).

**Table 3:** Unadjusted and adjusted association of energy-adjusted dietary inflammatory index (continuous) as a predictor on the 5-year incidence of metabolic syndrome (*n* = 568) and its five components separately as outcome variables in the logistic regression model (*n* = 2,252).

Variable	Unadjusted		Adjusted	
	OR (95%CI)	*P value	OR (95%CI)	*P value
MetS	1.26 (1.20, 1.33)	<0.001	1.29 (1.22, 1.36)	<0.001
low HDL-C	1.07 (1.04, 1.10)	<0.001	1.08 (1.04, 1.11)	<0.001
High BP	1.13 (1.08, 1.19)	<0.001	1.14 (1.09, 1.20)	<0.001
High TG	1.19 (1.13, 1.26)	<0.001	1.19 (1.13, 1.25)	<0.001
High FBS	1.12 (1.07, 1.18)	<0.001	1.12 (1.06, 1.18)	<0.001
High WC	1.13 (1.09, 1.16)	<0.001	1.17 (1.13, 1.21)	<0.001

*Logistic regression was used to investigate the adjusted and unadjusted association of the Energy-adjusted Dietary Inflammatory Index with a 5-year incidence of Metabolic Syndrome. Adjusted for age, gender, physical activity, and opium. MetS, metabolic syndrome; HDL-C, high-density lipoprotein cholesterol; BP, blood pressure; TG, triglyceride; FBS, fasting blood sugar; WC, waist circumference.

**Table 4:** The association of energy-adjusted dietary inflammatory index (continuous) with the incidence of metabolic syndrome and its component, considering body mass index (*n* = 2,252).

Variable	Low body mass index (< 25 kg/m^2^)		High body mass index (≥25 kg/m^2^)	
	OR (95%CI)	*P* value*	OR (95%CI)	*P* value*
MetS	1.42 (1.27, 1.60)	<0.001	1.13 (1.05, 1.21)	<0.001
low HDL-C	1.11 (1.05, 1.17)	<0.001	1.00 (0.93, 1.02)	0.306
High BP	1.07 (0.97, 1.19)	<0.001	1.09 (1.01, 1.18)	0.021
High TG	1.29 (1.17, 1.42)	<0.001	1.09 (1.01, 1.18)	0.026
High FBS	1.09 (0.99, 1.20)	0.072	1,96 (0.98, 1.14)	0.146
High WC	1.21 (1.12, 1.31)	<0.001	1.01 (0.96, 1.05)	0.832

*Logistic regression was used to investigate the adjusted association of the Energy-adjusted Dietary Inflammatory Index with a 5-year incidence of Metabolic Syndrome considering high or low body mass index. Adjusted for age, gender, physical activity, and opium. MetS, metabolic syndrome; HDL-C, high-density lipoprotein cholesterol; BP, blood pressure; TG, triglyceride; FBS, fasting blood sugar; WC, waist circumference.

Table 5 shows the association of E-DII with the diseases associated with MetS. The crude models showed that E-DII had a significant positive association with diabetes, hypertension, cardiovascular disease, MI, stroke, and fatty liver disease. Also, these associations remained significant after adjusting for confounding factors for each disease (addressed in Table 5). Then, Model 2 showed the role of MetS in these associations. Comparing Model 2 and Model 1 showed that adjusted for MetS decreased the OR for diabetes and hypertension, though they remained significant. Adjusting for MetS in the association of E-DII with MI decreased the OR and made the association insignificant. Remarkably, the OR of the association between E-DII and fatty liver disease increased after adjusting for MetS.

**Table 5:** The association of energy-adjusted dietary inflammatory index (continuous) with diabetes, hypertension, cardiovascular disease, myocardial infarction, stroke, and fatty liver disease, with and without considering the role of metabolic syndrome (*n* = 10,030).

Variable	Crude		Model 1		Model 2	
	OR (95%CI)	*p*-value	OR (95%CI)	*p*-value	OR (95%CI)	*p*-value
DM^a^	1.21 (1.18, 1.25)	<0.001	1.14 (1.10, 1.17)	<0.001	1.05 (1.02, 1.09)	0.004
HTN^b^	1.21 (1.18, 1.24)	<0.001	1.16 (1.13, 1.19)	<0.001	1.04 (1.01, 1.08)	<0.001
MI^c^	1.15 (1.07, 1.24)	<0.001	1.10 (1.01, 1.19)	0.022	1.08 (0.99, 1.18)	0.08
Stroke^d^	1.12 (1.03, 1.22)	0.009	1.04 (0.95, 1.15)	0.359	1.03 (0.94, 1.14)	0.44

^a^Diabetes Mellitus, Model 1 was adjusted for age, gender, hypertension, cardiovascular disease, fatty liver disease, and physical activity. Model 2 was adjusted for model 1 + MetS. ^b^Hypertension, Model 1 was adjusted for age, gender, diabetes, cardiovascular disease, stroke, fatty liver disease, physical activity, and smoking. Model 2 was adjusted for model 1 + MetS. ^c^Myocardial infarction, Model 1 was adjusted for age, gender, physical activity, diabetes, hypertension, occupation, and opium. Model 2 was adjusted for model 1 + MetS. ^d^Stroke, Model 1 was adjusted for age, gender, diabetes, hypertension, physical activity, and socioeconomic status. Model 2 was adjusted for model 1 + MetS.

## 4 Discussion

The present study was conducted on the FACS population to investigate the association between the diet’s inflammatory potential with prevalence and 5-year incidence of MetS. Our analysis indicated that a pro-inflammatory diet is significantly associated with a higher risk of developing MetS and its components in cross-sectional and prospective study designs. Furthermore, our results suggested that the association between E-DII and WC is more vital than other components. Interestingly, our study revealed that the pro-inflammatory diet increased the chance of MetS in both obese and normal-weight individuals. Still, the association is stronger among normal-weight participants than obese ones. This explains the prominent role of the inflammatory potential of diet in the progression to MetS among lean individuals and the prevention of MetS among obese people. In addition, we found that MetS mediated the association between E-DII and non-communicable diseases, including diabetes, MI, and hypertension. The pro-inflammatory diet increases the risk of these non-communicable diseases through MetS. Therefore, an anti-inflammatory diet decreases the risk of developing MetS and prevents cardiometabolic diseases.

In contrast to our findings, several studies reported no significant association between DII and MetS (29–33). There may be different reasons for their negative results. For instance, one study used a single 24-h recall, which did not provide sufficient information about participants’ dietary habits (22).

Similar to our study, previous observations showed that a pro-inflammatory diet (measured mainly by DII) had a significant positive association with MetS (27–31). A cross-sectional study of 1,992 Irish participants indicated that individuals with higher E-DII scores had a 37% increased risk of MetS (28). Additionally, Mazidi et al. revealed that individuals in the fourth quartile of E-DII had a 23% greater chance for MetS (29). The data from the Supplémentation en Vitamines et Minéraux AntioXydants (SU.VI.MAX) cohort consisting of 3,726 French participants with 13 years of follow-up showed that participants with the highest pro-inflammatory diet had a 39% greater chance for developing MetS (30). Furthermore, two cross-sectional studies in Iran showed a positive significant association between DII and MetS (31, 32).

A few systematic reviews and metanalysis attempt to evaluate the association of DII with MetS and its components (15, 33). A systematic review of studies found that participants in the pro-inflammatory diet had a significantly higher risk of MetS (OR = 1.23, 95%CI: 1.10–1.38) and its components except low HDL-C (33). Another systematic review and meta-analysis confirmed the association between DII and MetS (OR = 1.13, 95%CI: 1.03–1.25) but only found a significant association between DII and the two components of MetS, such as hypertension and hyperglycemia (15).

Our findings indicated that despite the strong association between E-DII and WC in cross-sectional analysis, the prospective analysis revealed that the TG level is the most critical component. Although there is an agreement with the positive association between the inflammatory potential of the diet and MetS with limited controversies, the most critical element of MetS that plays the central role in this association has remained controversial among previous studies. A prospective study in Korea indicated that higher DII was associated with higher risk for MetS and its components. The strongest association was between DII and HDL-C levels. However, the stratified analysis for sex showed that the association between DII and MetS was only consistent among women (34). A study of 100 Mexican individuals from infancy through adulthood found that the cumulative effect of the pro-inflammatory diet over the years increased the risk of MetS (35). A study of 8,180 participants from the 2007–2018 National Health and Nutrition Examination Survey (NHANES) indicated that individuals in the highest quartile of DII had a 1.59 times greater chance of MetS. Also, this study showed TG level had the strongest association with DII (*β* = 2.795) among MetS components (36). Also, a study in northern China showed that DII is significantly associated with MetS and its components. Wrist circumference had the strongest association with DII (37). Therefore, it is still unclear whether the dietary potential of diet had the most significant association with which MetS component. The findings of a previous study on FACS by Ariya et al. showed that adjusting the association of DII with MetS for BMI, as a well-known indicator of obesity (especially central obesity), did not remain significant anymore. Therefore, central obesity, the essential component of MetS, would be one of the most likely mediators of the association between DII and MetS, which matches our findings (38).

Higher E-DII is associated with obesity, which would mediate the effect of a pro-inflammatory diet on cardiometabolic diseases (39). Also, visceral obesity increases the pro-inflammatory biomarkers. Current evidence supports the idea that inflammation is a prime part of MetS pathophysiology. A pro-inflammatory state eventuates into insulin resistance and MetS components such as hypertension, dyslipidemia, and hyperglycemia. Adipose tissue modulates the inflammation by secreting adipokines. Adipose tissue produces TNF-α, IL-6, and monocyte chemoattractant protein-1 (MCP-1), which attract monocytes. Migrated monocytes produce more cytokines, disrupting insulin signaling by activating the intracellular kinases that cause serine phosphorylation of insulin receptor substrate-1 (IRS-1). Adiponectin is a hormone with anti-inflammatory effects excreted by fatty tissue, which regulates lipid and glucose metabolism. People with a higher proportion of adiposity have lower levels of adiponectin. Lower levels of adiponectin result in activating pro-inflammatory cytokines and decreased levels of anti-inflammatory cytokines like IL-10 (40, 41). As mentioned earlier, dietary patterns have a considerable role in developing MetS as well as modulating inflammation. Results from the HELENA study revealed that higher DII scores were significantly associated with higher levels of pro-inflammatory cytokines such as IL-1, IL-6, TNF-α, and vascular cell adhesion molecule (VACM), among European adolescents (42). Therefore, it is important to decrease the inflammatory potential of diet in younger individuals to prevent development of MetS and its related diseases in adulthood. Also, previous studies showed DII was significantly associated with C-reactive protein level which demonstrates the inflammatory status (43, 44). Moreover, increased saturated fatty acids (SFA), which is one of the pro-inflammatory components of DII, lead to the activation of toll-like receptors (TLR) which facilitate insulin resistance by activating intracellular kinases. Additionally, activated kinases upregulate the nuclear factor kappa-B (NF-kB) which results in interfering insulin signaling through increased amounts of pro-inflammatory cytokines (40). Taken together, the pro-inflammatory diet has a paramount contribution in developing MetS through modulating inflammatory cytokines. Additionally, people with obesity are in a higher inflammatory state than lean individuals because of adipose tissue dysfunction (45). Therefore, an equal inflammatory potential of diet has a greater effect on the inflammatory state of lean individuals explaining the stronger association between E-DII and MetS among non-obese participants.

Strengths and limitations. The present study had several strengths. This study was conducted in a longitudinal design with 5 years of follow-up in a large-population cohort study. Furthermore, this study used the data from FACS, a validated comprehensive study of more than 10,000 people with detailed information about the participants, which allowed us to use multi-variable adjustments in the analysis. In addition, E-DII was used to estimate the diet’s inflammatory potential, which is more accurate than DII.

Also, our study had some limitations. The follow-up was conducted for a proportion of the participants included in the cross-sectional phase. Besides, our FFQ contained 31 parameters of 45 anti- or pro-inflammatory parameters of E-DII.

## 5 Conclusion

The present study revealed the significant association of the diet’s inflammatory potential with the prevalence and 5-year incidence of MetS and its components, especially central obesity, among adult population. Therefore, obesity and a pro-inflammatory diet would have a two-way synergic effect, increasing inflammation and provoking MetS and its further complications. Also, our findings indicated that E-DII would have a significant role in the progression of MetS among non-obese participants. Moreover, MetS mediates the association between E-DII and cardiometabolic diseases. In conclusion, controlling the inflammatory potential of diet would be a promising way to prevent MetS and cardiometabolic diseases.

## Data availability statement

The raw data supporting the conclusion of this article will be made available by the authors, subject to approval from the Ethics Committee of Fasa University of Medical Sciences.

## Ethics statement

The studies involving humans were approved by Ethics Committee of Fasa University of Medical Sciences (IR.FUMS.REC.1401.162). The studies were conducted in accordance with the local legislation and institutional requirements. The participants provided their written informed consent to participate in this study.

## Author contributions

HP: Conceptualization, Writing – original draft. MS: Investigation, Writing – original draft. MK: Writing – review & editing. MF: Data curation, Funding acquisition, Investigation, Supervision, Writing – review & editing. FV: Writing – review & editing. AD: Data curation, Investigation, Validation, Writing – review & editing. RH: Conceptualization, Investigation, Supervision, Writing – review & editing. MN: Conceptualization, Formal analysis, Funding acquisition, Methodology, Software, Supervision, Writing – review & editing. JH: Writing – review & editing.
