# Supplementary material for: The association between energy-adjusted dietary inflammatory index and metabolic syndrome and its mediatory role for cardiometabolic diseases: a prospective cohort study
Source: Front Nutr. 2024 Aug 5;11:1429883. doi: 10.3389/fnut.2024.1429883 (PMC11330808; doi:10.3389/fnut.2024.1429883)
Supplement: Supplementary file 1 [file Table_1.docx]

**Supplementary 1.** Nutrients intake of participants according to Metabolic Syndrome (n = 10025).

|  | Total | | Metabolic Syndrome | | | | P value |
| --- | --- | --- | --- | --- | --- | --- | --- |
|  |  |  | No | | Yes | |  |
|  | Mean | Standard Deviation | Mean | Standard Deviation | Mean | Standard Deviation |  |
| Vitamin B12 (µg) | 3.13 | 3.51 | 3.28 | 3.70 | 2.65 | 2.76 | <0.001 |
| Vitamin B6 (mg) | 0.92 | 0.47 | 0.94 | 0.47 | 0.87 | 0.44 | <0.001 |
| Beta-Carotene (µg) | 872 | 976 | 890 | 1002 | 815 | 885 | <0.001 |
| Carbohydrate (g) | 450 | 202 | 457 | 205 | 427 | 190 | <0.001 |
| Caffeine (g) | 0.04 | 0.19 | 0.05 | 0.12 | 0.03 | 0.18 | 0.011 |
| Cholesterol (mg) | 276 | 165 | 288 | 169 | 237 | 147 | <0.001 |
| Energy (kcal) | 2780 | 1140 | 2835 | 1158 | 2603 | 1063 | <0.001 |
| Total fat (g) | 68.8 | 34.3 | 70.9 | 35.3 | 62.3 | 30.0 | <0.001 |
| Fiber (g) | 20.5 | 10.2 | 20.6 | 10.3 | 20.0 | 9.7 | 0.008 |
| Folic Acid (µg) | 180 | 110 | 183 | 112 | 170 | 106 | <0.001 |
| Fe (mg) | 16.6 | 8.2 | 16.9 | 8.4 | 15.6 | 7.6 | <0.001 |
| Mg (mg) | 168 | 86 | 172 | 87 | 158 | 81 | <0.001 |
| MUFA (g) | 20.2 | 11.6 | 20.9 | 12.0 | 18.1 | 10.0 | <0.001 |
| Niacin (mg) | 8.87 | 5.57 | 9.06 | 5.66 | 8.23 | 5.23 | <0.001 |
| n-3 fatty acid (g) | 0.40 | 0.44 | 0.40 | 0.48 | 0.38 | 0.29 | 0.042 |
| n-6 fatty acid (g) | 3.00 | 3.47 | 3.02 | 3.72 | 2.97 | 2.53 | 0.536 |
| Protein (g) | 91.7 | 39.9 | 93.5 | 40.6 | 85.7 | 37.2 | <0.001 |
| PUFA (g) | 8.53 | 5.37 | 8.73 | 5.64 | 7.92 | 4.36 | <0.001 |
| Riboflavin (mg) | 0.91 | 0.50 | 0.93 | 0.51 | 0.84 | 0.45 | <0.001 |
| Se (µg) | 49.0 | 30.2 | 51.0 | 30.8 | 42.8 | 27.3 | <0.001 |
| Thiamin (mg) | 0.58 | 0.40 | 0.59 | 0.39 | 0.56 | 0.42 | <0.001 |
| Trans fat (g) | 0.13 | 0.15 | 0.13 | 0.16 | 0.11 | 0.13 | <0.001 |
| Vitamin A (RAE) | 392 | 345 | 407 | 358 | 343 | 294 | <0.001 |
| Vitamin C (mg) | 71.8 | 75.0 | 71.6 | 74.0 | 72.4 | 78.0 | 0.666 |
| Vitamin D (µg) | 1.33 | 1.06 | 1.39 | 1.10 | 1.13 | 0.92 | <0.001 |
| Vitamin E (mg) | 3.70 | 2.84 | 3.77 | 2.94 | 3.50 | 2.53 | <0.001 |
| Zn (mg) | 5.88 | 2.88 | 6.04 | 2.93 | 5.39 | 2.66 | <0.001 |
| Garlic (g) | 0.35 | 2.24 | 0.40 | 2.54 | 0.19 | 0.66 | <0.001 |
| Onion (g) | 78.2 | 76.8 | 84.1 | 82.5 | 59.0 | 50.7 | <0.001 |
| Peppers (g) | 9.93 | 24.2 | 11.2 | 26.7 | 5.9 | 11.9 | <0.001 |
| Saturated fat (g) | 25.9 | 11.3 | 27.6 | 11.6 | 20.4 | 8.19 | <0.001 |

**Note:** Quantitative variables are presented as mean ± standard deviation (SD). Independent T-test compared the food parameters intake between participants with and without Metabolic Syndrome. P-value <0.05 was considered as statistically significant.

**Abbreviations:** Fe, Iron; Mg, Magnesium; MUFA, monounsaturated fatty acids; Se, Selenium; PUFA, polyunsaturated fatty acids; RAE, retinol activity equivalent; Zn, Zinc.
